# Prediction of stroke risk based on left atrial appendage morphology: from pareidolia to artificial intelligence

**DOI:** 10.1007/s10554-021-02307-y

**Published:** 2021-06-29

**Authors:** Till F. Althoff, Lluís Mont

**Affiliations:** 1 Department of Cardiology and Angiology, Charité Campus Mitte, Charité – University Medicine Berlin, Charitéplatz 1, 10117 Berlin, Germany; 2 DZHK (German Centre for Cardiovascular Research), partner site Berlin, Berlin, Germany; 3 Hospital Clínic Atrial Fibrillation Unit (UFA), Arrhythmia Section, Cardiovascular Institute (ICCV), Hospital Clínic, University of Barcelona, C/Villarroel No 170, 08036 Barcelona, Spain; 4 Institut D’Investigacions Biomèdiques August Pi I Sunyer (IDIBAPS), Barcelona, Catalonia Spain; 5 Centro de Investigación Biomédica en Red Cardiovascular (CIBERCV), Madrid, Spain

Stroke is the most devastating complication of atrial fibrillation (AF). Even though anticoagulation has proven to be an effective prophylactic treatment, its benefits must be balanced against an increased risk of bleeding [1]. Therefore, risk stratification is essential in patients with AF. In current clinical practice, stratification of patients according to stroke risk is merely based on the CHA_2_DS_2_-VASc score [1]. However, even in patients with low or moderate risk according to the CHA_2_DS_2_-VASc score, for whom guidelines do not recommend oral anticoagulation, strokes do occur [2, 3]. Thus, there is an unmet need to refine current predictive models to better define the individual risk and select patients that may benefit from anticoagulation despite low CHA_2_DS_2_-VASc score (0 or 1).

As the left atrial appendage (LAA) may play an important role in the etiology of AF-related cardioembolic stroke, it is well conceivable that LAA morphology impacts stroke risk [4]. In fact, Di Biase et al. demonstrated that an LAA shape that they perceived as chicken wing-like was associated with a significantly lower risk of stroke compared to other morphologies they perceived as cauliflower-, windsock- or cactus-shape [5]. This tendency to associate complex structures with familiar objects has been termed pareidolia and reflects the unique capability of the human brain to recognise patterns in visual stimuli. However, just like seeing animals in clouds, classifying LAAs into chicken wing, cauliflower, cactus and windsock based on pareidolia is somewhat arbitrary and obviously highly subjective. Moreover, while the predictive value implicates that the typical features of an LAA perceived as chicken wing-like comprise morphological determinants of decreased stroke risk, these arbitrarily defined features are unlikely to be fully congruent with the truely risk-defining factors. Thus, with the current LAA-classification by Di Biase et al., we are rather dealing with a probabilistic model, where the LAA morphology perceived as chicken wing-like is by chance more likely to comprise underlying factors that determine a lower risk.

More recently, better defined quantitative LAA-related parameters have been proposed, like high LAA takeoff, angle of LAA bend, ostium size, LAA size or trabecularisation [2, 6–8]. However, evidence on the predictive value of these parameters and LAA morphology is conflicting [5, 9, 10], and to date no predictive parameter or model related to LAA morphology has been established in clinical practice [1]. While simple quantitative parameters like the bend angle or size of the LAA are more objective and reproducible, they are likely to fall short of the complexity of the 3D geometry and the underlying stroke-promoting factors. Moreover, they are based on a priori hypotheses and are thus biased. On the other hand, as outlined above a rather arbitrary qualitative classification of LAA 3D morphologies based on pareidolia or human pattern recognition is obviously limited by substantial cognitive bias and the processing capacity of the human brain.

Artificial intelligence (AI) with its capability to learn discriminative features automatically and to approximate even very complex nonlinear relationships, has the potential to overcome this quandary. Machine learning approaches of pattern recognition along with the computing power inherent to AI have already started to excel human thinking in the assessment and interpretation of complex data [11]. Against this background, coupling of AI-recognised patterns with clinical outcome data may yield powerful biomarkers.

In this issue of the journal Bieging et al. used an algorithm based on artificial intelligence and machine learning in a broader sense, to generate a predictive model for stroke risk in patients with atrial fibrillation based on LA and LAA morphology. Their method implies particle-based modeling, a statistical analysis that automatically computes the 3D geometry through surface points that correspond to specific anatomical locations. Principal component analysis of these data sets was applied to automatically identify quantitative patterns of the 3D geometry that accounted for interindividual variability and subsequently to determine which of these patterns would predict clinical outcome. Although the details of the algorithm are not fully disclosed, such an AI-based approach has the potential to capture the full degree of complexity of the LA and LAA 3D geometries and their interplay with hemodynamics. It further allows for unbiased recognition of complex patterns, thus providing an objective quantitative assessment.

Indeed, three parameters accounting for LAA morphological variability, but no single LA parameter, were identified that predict stroke risk independent of established risk factors, and the combination of all three LAA parameters substantially improved the predictive capacity of the CHA_2_DS_2_-VASc score. Generally-speaking, a broader, shorter LAA body with the tip superior to the LAA ostium was found to imply a greater stroke risk. This may be roughly in line with previous data pointing into a similar direction. However, given the computational approach integrating the 3D geometries of many LAAs in an unbiased manner, the parameters identified by Bieging et al. are more likely to comprise the true morphological risk determinants, and the features yielded by this approach may better define individual stroke risk, particularly in a model that combines the three distinct parameters. Finally, these quantitative parameters allow for objective interindividual comparisons – a prerequisite to establish a predictive model as a standardised biomarker.

While the risk model proposed here needs to be refined and validated in larger prospective cohorts, there is no doubt that approaches employing AI, like the one presented by Bieging et al., bear a great potential to improve current predictive models and risk stratification of patients with atrial fibrillation. Obviously, this is a great advancement from previous approaches that more or less abritrarily defined LAA-related risk factors based on hypothetical assumptions and pareidolia.
